# Not Your Everyday FCD: Imaging Findings of Focal Cortical Dysplasia Type 1

**DOI:** 10.5334/jbsr.2710

**Published:** 2022-05-05

**Authors:** Stijn Marcelis, Stephanie Vanden Bossche, Sven Dekeyzer

**Affiliations:** 1 AZ Nikolaas, BE; 2 UZA, BE

**Keywords:** epilepsy, focal cortical dysplasia, FCD type 1

## Abstract

**Teaching Point:** The imaging findings of focal cortical dysplasia type 1 are less well-known than focal cortical dysplasia type 2 and consist of increased white matter signal intensity and blurring of the gray-white matter in a large region of the brain, with associated segmental or lobar brain atrophy.

## Case History

**Figure 1:**
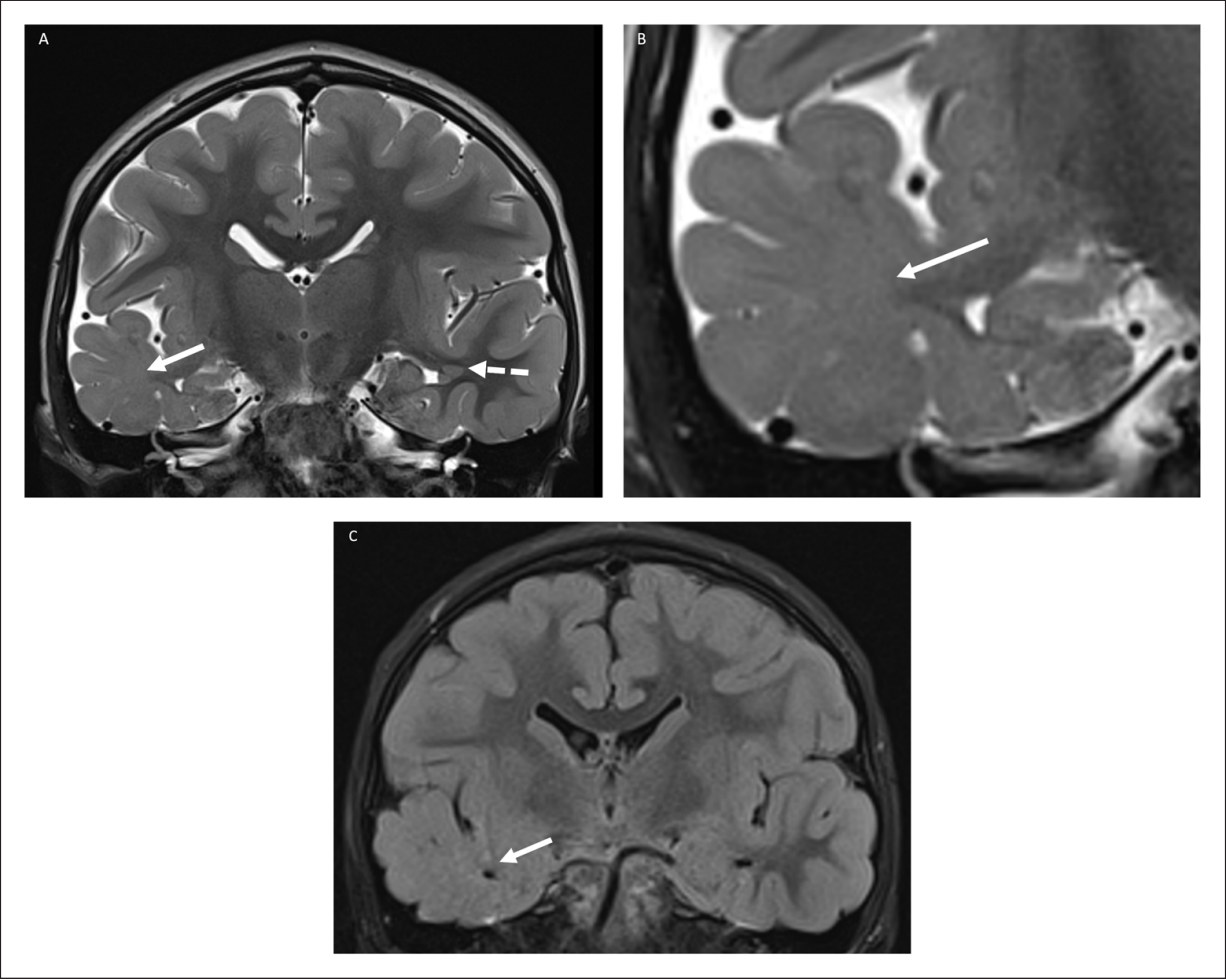


A six-year-old girl was referred to a tertiary center because of a status epilepticus. The patient was known to have microcephaly and neuromotor retardation due to a 6q-terminal deletion syndrome. She had an extensive history of drug-resistant complex partial seizures. Magnetic resonance imaging (MRI) of the brain was performed and showed callosal and pontine dysgenesis, findings known to be associated with 6q-terminal deletion syndrome. Additionally, several periventricular nodular heterotopias were seen (***Figure 1A***, white stippled arrow), as well as a decreased volume of the right temporal lobe with diffusely increased signal of the subcortical white matter on all sequences and complete blurring of the gray-white matter junction (***Figure 1A–C***, white arrows), the typical imaging findings of focal cortical dysplasia type 1.

## Comment

Focal cortical dysplasia (FCD) is a group of disorders characterized by cortical architectural abnormalities with or without the presence of abnormal neurons. FCD is the most frequent cause of medically refractory epilepsy in children.

Three types of FCD are recognized. FCD type 1 is characterized by abnormal cortical layering without abnormal cells. FCD type 2 consists of abnormal cortical layering with cytological abnormalities (dysmorphic neurons and balloon cells). FCD type 3 is an FCD type 1 with another abnormality such as hippocampal sclerosis, glioneuronal tumor, vascular malformations, or acquired lesions (ischemic infarction or encephalitis).

The best known FCD subtype is FCD type 2. The imaging findings of FCD type 2 consist of cortical thickening, typically around the bottom of a sulcus, with blurring of the gray-white matter differentiation, and with a characteristic “transmantle sign”.

FCD type 1 can be a more difficult diagnosis. Typical imaging findings are a moderately increased T2/FLAIR-signal and decreased T1-signal of the white matter with blurring of the gray-white matter junction and prominent segmental or lobar hypoplasia of the affected region. FCD type 1 can be subdivided in type 1a (involves the temporal lobe) and type 1b (more often seen extratemporal). FCD type 1 can remain radiologically occult in up to 12% of patients, hence high-resolution imaging is necessary, preferentially at a higher field strength (3T), to maximize the detection rate.

FCD type 1 can occur in isolation or be associated with other brain lesions. FCD type 1 associated with hippocampal sclerosis or glioneural tumors are now considered FCD type 3. Other malformations that may be associated with FCD type 1 are periventricular nodular heterotopia and hamartomas [1].
